# The COVID‐19 Pandemic and Its Influence on Patients With Myotonic Dystrophy Type 1: Lessons Learned

**DOI:** 10.1002/brb3.70162

**Published:** 2024-11-17

**Authors:** Vera E. A. Kleinveld, Johanna E. Bruijnes, Samuel Labrecque, Danielle E. G. Jeurissen‐Bekkering, Catharina G. Faber, Corinne G. C. Horlings

**Affiliations:** ^1^ Department of Neurology Medical University of Innsbruck Innsbruck Austria; ^2^ Department of Neurology, School of Mental Health and Neuroscience Maastricht University Medical Centre+ Maastricht the Netherlands

**Keywords:** COVID‐19, muscular dystrophies, myotonic dystrophy, pandemic

## Abstract

**Introduction**: Myotonic dystrophy type 1 (DM1) patients might represent a high‐risk population for severe COVID‐19 disease, as cardiopulmonary symptoms are part of the clinical spectrum of DM1. The COVID‐19 pandemic may have impacted DM1 patients. We aimed to determine the effect of the COVID‐19 pandemic on DM1 patients to guide management strategies in possible future pandemics.

**Methods**: Data on the presence of a COVID‐19 infection were retrieved from 195 DM1 patients in the MYODRAFT database. Between August 12 and October 4, 2021, 82 patients and proxies filled out a questionnaire on COVID‐19 symptoms, well‐being, and organization of care. Data were compared to prepandemic data.

**Results**: A total of 18 patients had COVID‐19 (13 confirmed, 5 probable infections). The prevalence of COVID‐19 in our cohort was 9.2%, which was lower than in the Dutch population (11.5%). Four patients (22.2%) were hospitalized due to a COVID‐19 infection, which was higher than in the Dutch population (3.6%). Two infected patients died. A high rate of canceled appointments was reported. Patients reported no change in physical functioning during the pandemic, whereas proxies reported a deterioration in mental and physical well‐being of patients.

**Conclusions**: The prevalence of COVID‐19 infections was not higher in DM1 patients than in the general population, but DM1 patients are more susceptible to complicated disease when infected. Longitudinal data on patient‐reported physical functioning suggest that the COVID‐19 pandemic and the pandemic management strategies implemented did not influence the course of disease in DM1 patients, and similar strategies can be re‐used in comparable situations.

## Graphical abstract

The occurrence and effects of COVID‐19 in a myotonic dystrophy type 1 (DM1) population (*n* = 195) were studied. We found DM1 patients were more susceptible to complicated COVID‐19 disease. However, patient‐reported longitudinal data on physical functioning suggested that the COVID‐19 pandemic did not influence the course of disease in DM1.

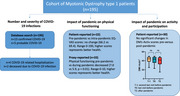

## 1 Introduction

Myotonic dystrophy type 1 (DM1) is the most common inherited form of muscular dystrophy in adults (Gutiérrez Gutiérrez et al. 2020). It has a wide phenotypic spectrum, and great disease variability between patients exists, ranging from asymptomatic disease to severe disabilities. DM1 is characterized by progressive neuromuscular disease and causes myotonia and muscle weakness, but it also affects other organs as well, leading to, for example, heart conduction disorders and respiratory weakness (Gutiérrez Gutiérrez et al. 2020). As respiratory muscle weakness and cardiopulmonary dysfunction are common in patients with DM1, patients might be a high‐risk population for respiratory infections, including severe COVID‐19 (Dhont et al. 2021).

Not only deterioration due to a COVID‐19 infection can be expected in neuromuscular patients but also the governmental restrictions imposed due to the COVID‐19 pandemic, for example, the prohibition of ambulant physical and occupational therapy, closing of sports facilities, and the restrictions on social contacts, impact physical and mental health. In chronic neurological populations, for example, Parkinson's disease, it was seen that the pandemic had negative effects on the physical and mental health of patients. Patients were more prone to psychological disturbances attributed to the imposition of social restrictions and fear of getting infected (Brooks, Weston, and Greenberg 2021; Salari et al. 2020). Negative effects on mental well‐being were demonstrated in patients with multiple sclerosis as well (Stojanov et al. 2020; Uhr, Rice, and Mateen 2021). There is evidence of negative effects on DM1 patients (Eichinger et al. 2021). Major challenges among DM1 patients, reported during the pandemic, included obtaining treatment and managing stress. DM1 patients reported significantly higher levels of stress during the COVID‐19 pandemic, and interruptions in health care delivery (canceled or rescheduled health care visits) were common (Eichinger et al. 2021). The governmental restrictions may also have a greater impact on DM1 patients than on the general population, whereas cognitive deficits, depression, apathy, and anxiety are common symptoms of DM1 and part of the clinical spectrum (Antonini et al. 2006; Gallais et al. 2015; Miller et al. 2021). Pre‐existing apathy already reduces social interaction and physical activity (van der Velden et al. 2019; Van Heugten et al. 2018).

The primary objective of this study is to gain insights into the severity of COVID‐19 infection in a large cohort of DM1 patients, as represented by hospitalization rates and mortality. As secondary objective, the influence of the pandemic on physical and mental well‐being of DM1 patients will be reviewed. This could serve as a footing in the decision‐making process of the organization of care for DM1 patients in possible future pandemics or comparable situations.

## 2 Methods

### 2.1 Design

This is a single‐center, retrospective, observational study at the Maastricht University Medical Centre+ (MUMC+), the Netherlands. Data were retrieved from the observational DM1: Dutch Registry and Follow‐up Study (MYODRAFT study), a patient registry and natural history database of DM1 patients (start of data collection: March 2017). All included patients were aged 18 years or older, had a genetically confirmed DM1 diagnosis, and provided written informed consent for participation in the MYODRAFT study. For the additional COVID‐19‐related questionnaire, additional written informed consent was obtained. The study was performed in accordance with the Helsinki Declaration of 1975 and approved by the medical ethics committee of the Medical University Centre Maastricht, the Netherlands (no. 16‐4‐001).

### 2.2 Definition of Time‐Points

March 15, 2020 was defined as the start of the pandemic, as this corresponds with the start of the first lockdown in the Netherlands. March 1, 2022 was defined as the end of the pandemic, as from then on, the last COVID‐19‐related regulations were abolished.

### 2.3 Data Extracted From the MYODRAFT Database

Patients included in the MYODRAFT database attend an annual visit. Data extracted from the MYODRAFT database included data on the diagnosis, demographic data, and data on disease progression. For the current study, all consecutive patients who joined the MYODRAFT study until August 12, 2021 were included.

In the MYODRAFT database, DM1 progression is monitored through the Rasch‐Built Myotonic Dystrophy Type 1 Activity and Participation Scale (DM1‐Activ^c^ scale). This is a 25‐item unidimensional scale of activity and participation (Hermans et al. 2015). The most recent DM1‐Activ^c^ score before the COVID‐19 pandemic was assessed and compared to the DM1‐Activ^c^ score at the visit before to assess how this score changes over time in a period not affected by the pandemic. Subsequently, we compared the most recent prepandemic DM1‐Activ^c^ score to the first score that was recorded after the pandemic started to evaluate disease progression during the pandemic. The intrapandemic and prepandemic scores were compared with scores obtained after the pandemic.

### 2.4 Scoring of the DM1‐Activ^c^ Survey

The standard DM1‐Activ^c^ scoring‐algorithm was used (Draak et al. 2014; Hermans et al. 2015), in which the minimally clinically important difference‐related standard error (MCID‐SE) score is computed for each examined patient separately by using their personal change (personal‐location at time‐point *x* − personal‐location at time point *y*). The individual change scores are then divided by their corresponding change in standard error (SE_diff_):

MCID‐SE = (personal‐location at second follow‐up − personal‐location at first follow‐up)/SE_diff_.

Subsequently, the calculated MCID‐SE findings can be divided into the following subgroups:

- Subgroup 1 (clinically important improvement): MCID‐SE ≥ 1.96;
- Subgroup 2 (clinically unimportant improvement): 0 < MCID‐SE < 1.96;
- Subgroup 3 (no change): MCID‐SE = 0;
- Subgroup 4 (clinically unimportant deterioration): −1.96 < MCID‐SE < 0; and
- Subgroup 5 (clinically important deterioration): MCID‐SE ≤ −1.96).

### 2.5 Data Obtained From the COVID‐19 Questionnaire

All included patients were asked to complete an additional questionnaire (Appendix). Questionnaires could be completed between August 12 and October 4, 2021. This COVID‐19 questionnaire concerned the occurrence of a COVID‐19 infection, COVID‐19 symptoms, necessity of hospital admission due to COVID‐19 infection, current health status, and healthcare appointments. COVID‐19 diagnosis was confirmed when patients reported a SARS‐CoV‐2 polymerase chain reactive (PCR)‐positive nasopharyngeal swab. Probable COVID‐19 was assessed by the presence of suggestive clinical symptoms, such as fever, cough, dyspnea, or anosmia/dysgeusia, in combination with close contact with an infected patient in the period preceding the start of symptoms. Part of the additional questionnaire was the European Quality of Life five‐dimension (EuroQol‐5D) survey. This is a patient‐reported descriptive system on five domains (mobility, self‐care, usual activities, pain/discomfort, and anxiety/depression). One part of the EuroQol‐5D survey is the EuroQol five dimensions and five levels (EQ‐5D‐5L), in which a higher score on one of the five domains corresponds with more limitations in that domain. The patient is asked to indicate the level of perceived problems per domain on a 1–3 Likert scale (range: −0.59 to 1, with higher scores indicating better health state). The EuroQol Visual Analog Scale (EQ‐VAS) rates the patients' general health on a 0–100 scale (EuroQol). A higher score represents a better health status. EQ‐VAS scores were compared to data from the MYODRAFT database before the start of the COVID‐19 pandemic. In addition, proxies were asked to retrospectively score the physical functioning and mental well‐being of the patient prepandemic and intrapandemic on a numeric 1–10 scale, with a higher score indicating better physical functioning or higher appreciation of well‐being.

### 2.6 Statistical Analysis

Variables were described in percentages with ranges, and in mean and standard deviation for normally distributed parameters and with median and interquartile range for non‐normally distributed parameters. Shapiro–Wilk tests were used to assess the distribution of the data. Mann–Whitney *U* test and Wilcoxon rank tests were used for comparisons between unpaired and paired non‐normally distributed variables (which included EQ‐VAS scores, DM1‐Activ^c^ scores, and proxy‐reported assessment of physical functioning and mental well‐being), respectively. Multiple linear regression analysis was performed on the EQ‐VAS scores in relation to EQ‐5D‐5L scores. Friedman test was used to test for differences between non‐normally distributed repeated measures, and post hoc tests were performed using Wilcoxon rank tests. Significance was defined as *p* < 0.05. Data are presented as mean ± standard deviation unless stated otherwise.

## 3 Results

**FIGURE 1:**
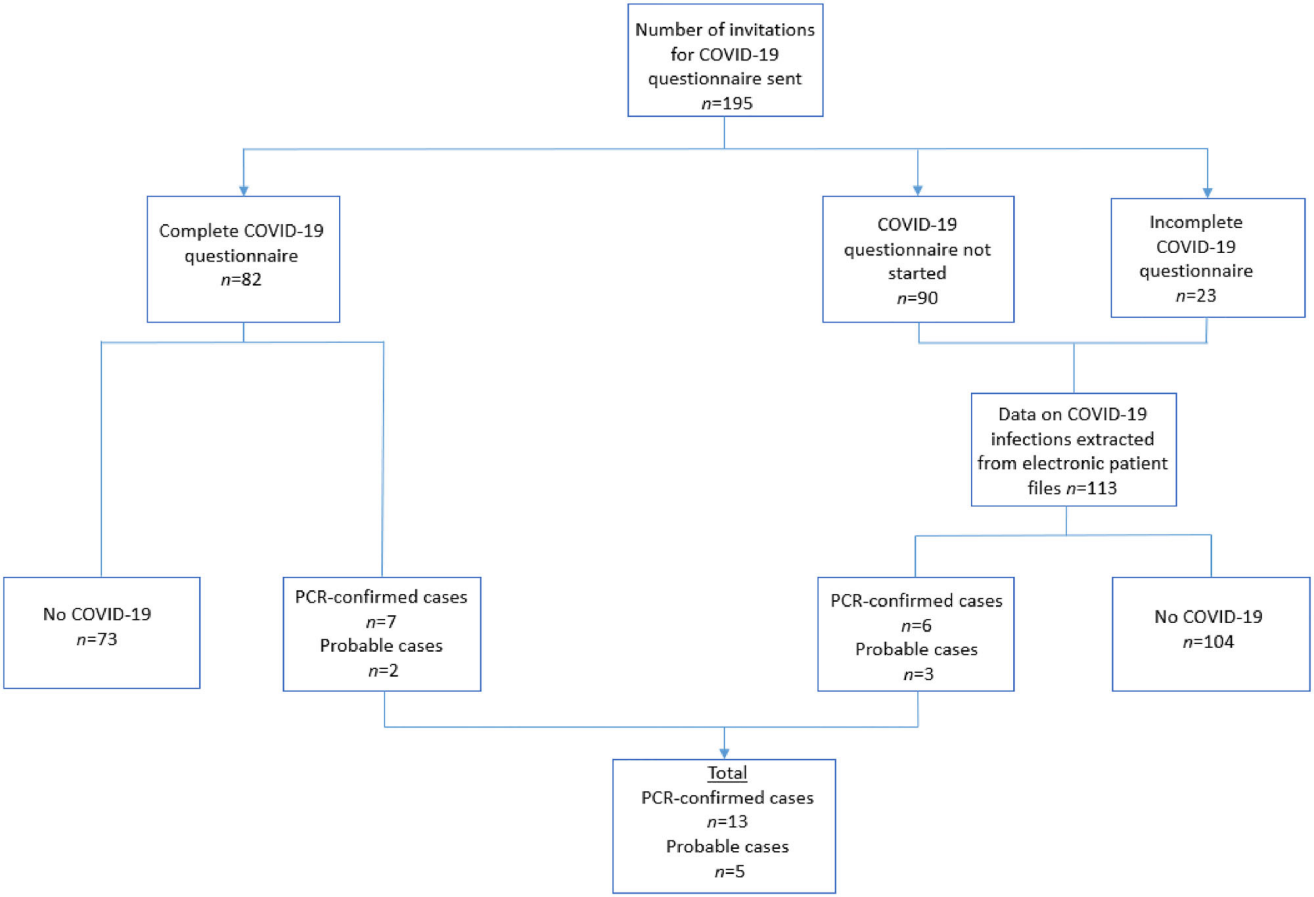
Algorithm of the study. PCR, polymerase chain reaction.

A total of 195 patients in the MYODRAFT database were included, and data were extracted. An invitation to participate in the questionnaire was sent to the included patients. Eventually, 82 patients completed the additional COVID‐19 questionnaire, and 23 patients partially completed the survey (see Figure 1). Demographic data are listed in Table 1.

**TABLE 1:** Sociodemographic data of DM1 patients.

Demographic data	Number or mean
Total number (%)	82 (100.0%)
Female sex, number (%)	41 (50.0%)
BMI, mean, kg/m^2^ (standard deviation)	25.6 (± 4.5)
Vaccinated against COVID‐19, number (%)	76 (92.7%)

Abbreviations: BMI, body mass index; SD, standard deviation.

### 3.1 COVID‐19 Infections in DM1 Patients

A total of 18 out of the 195 DM1 patients had a PCR‐confirmed or probable COVID‐19 infection, of which *n* = 13 confirmed and *n* = 5 probable. The prevalence of COVID‐19 in our DM1 population is 9.2% (CI: 0.056–0.142). Nine of the 18 DM1 patients with confirmed or probable COVID‐19 infection completed the COVID‐19 questionnaire on related symptoms, diagnostics, and treatment. All of these patients went through a symptomatic infection. Most frequently, patients reported increased fatigue, followed by cough. Reported symptoms are listed in Table 2.

**TABLE 2:** Reported symptoms after COVID‐19 infection in < 14 days after onset of the first symptom.

Symptom	Number of patients (*n* = 18)
Increase in fatigue	7
Cough	6
Fever (> 38°C)	5
Loss of taste/smell	5
Increased muscle strain	5
Headache	4
Sore throat	3
Increase in muscle weakness	3
Sinusitis	3
Increase in GI symptoms	2
Dyspnea	1

Abbreviations: DM1, Myotonic Dystrophy type 1; GI, gastrointestinal.

Four patients (22.2% of those infected) needed hospitalization due to the COVID‐19 infection. Two patients died as a result of a COVID‐19 infection. Both patients that died were overweight (BMI 26.5 and 35.9) and had a pre‐existing restrictive lung function disorder. One of the deceased patients had cardiac risk factors as well (first‐degree AV‐block and cardiac ischemia).

### 3.2 Healthcare Organization for DM1 Patients During the Pandemic

A total of 19 out of the 82 patients reported that 123 (mean per patient 3.62, range: 1–20) appointments with a health care professional were canceled. Of these appointments, 63.6% were canceled at the initiative of the health care professional. The remaining appointments were canceled by DM1 patients themselves because of fear of getting infected with COVID‐19. Canceled appointments concerned mostly appointments with medical specialists other than neurologists (40.0%), physiotherapists (30.7%), and neurologists (23.3%).

### 3.3 Well‐Being and Physical Functioning During the Pandemic

For 22 patients, prepandemic EQ‐VAS scores were available. There was no significant change in EQ‐VAS scores comparing prepandemic and intrapandemic EQ‐VAS scores (66.2 vs. 69.4 points, respectively, *p* = 0.299). According to the EQ‐5D‐5L scores, slight (34% of patients), moderate (15% of patients), and severe (4% of patients) problems in usual activities were reported. In addition, 29% of the patients reported being slightly anxious, 9% being moderately anxious, and 2% being severely anxious. Slight pain/discomfort was reported in 37% of patients, moderate pain/discomfort in 8%, and severe pain/discomfort in 12%. Regression analysis on the EQ‐5D‐5L in relation to the EQ‐VAS showed that more limitations in daily activities (*β = *−0.312, *p* < 0.05), more fear/anxiety (*β = *−0.214, *p* < 0.01), and more pain/discomfort (*β = *−0.194, *p* < 0.05) negatively affected EQ‐VAS scores.

The physical functioning of the patient was assessed by 33 caregivers and was evaluated as having decreased significantly during the pandemic (7.0 prepandemic vs. 5.9 during the pandemic, *p* < 0.01). Similarly, mental well‐being of the patient assessed by caregivers also decreased significantly (7.4 prepandemic vs. 6.8 during the pandemic, *p* < 0.01).

### 3.4 Activities of Daily Living Before, During and After the Pandemic

**FIGURE 2:**
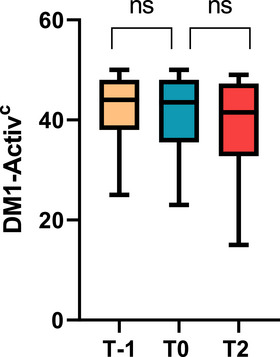
DM1‐Activ^c^ scores in patients before and after the pandemic. DM1‐Activ^c^, Rasch‐Built Myotonic Dystrophy Type 1 Activity and Participation Scale; T−1: second‐last assessment before the pandemic; T0: last assessment before pandemic; T2: first assessment after pandemic. Mean 14.6 (±0.87) months between T−1 and T0 and 39.2 (±1.04) months between T0 and T2.

**FIGURE 3:**
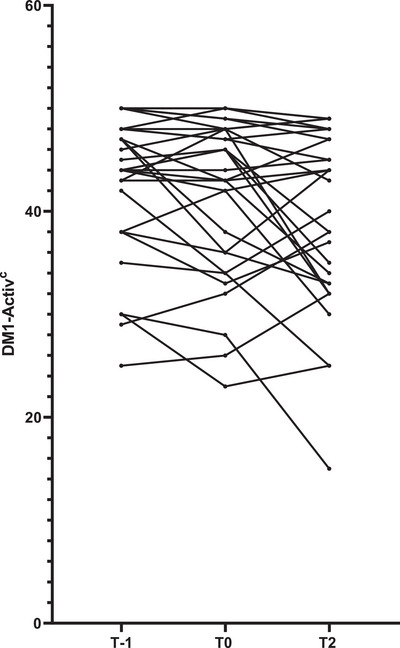
Longitudinal DM1‐Activ^c^ scores. DM1‐Activ^c^, Rasch‐Built Myotonic Dystrophy Type 1 Activity and Participation Scale; T0: last assessment before pandemic; T2: first assessment after pandemic. Mean 14.6 (±0.87) months between T−1 and T0 and 39.2 (±1.04) months between T0 and T2.

DM1‐Activ^c^ scores were collected twice before the pandemic (second‐last assessment before the pandemic, T−1 and last visit before the pandemic, T0) and once after the pandemic (T2) in 30 patients. Even though Friedman test revealed a significant difference among the assessments (*p* < 0.01), post hoc analyses revealed no significant difference between T−1 and T0 (*p* = 0.206) nor between T0 and T2 (*p* = 0.127) and T−1 and T2 (*p* = 0.051), see Figures 2 and 3. In times before the pandemic (from T−1 to T0), the mean rate of decrease in DM1‐Activ^c^ score was 0.09 ± 0.05 points/month, whereas it was 0.06 ± 0.03 points/month during the pandemic (from T0 to T2), which was not statistically different (*p* = 0.943).

Before the pandemic (T−1 vs. T0), 3 of 30 patients met the criteria for clinical deterioration, whereas 1 of the 30 patients improved. During the pandemic (T1), compared to the last visit before the pandemic (T0), two of the three patients that deteriorated before remained stable, whereas one patient improved. In addition, three other patients met the criteria for clinical deterioration, and one improved. After the pandemic (T2), compared to during the pandemic (T1), five patients deteriorated (of which none of the patients deteriorated during the pandemic), whereas one met the criteria for clinical improvement.

## 4 Discussion

Our data demonstrate that DM1 patients are at higher risk for complicated disease. In all cases, COVID‐19 infection resulted in symptomatic infection. The rate of hospitalization (22.2%) observed was high compared to the hospitalization rate in the general Dutch population (3.77%) (Epidemiologische situatie van SARS‐CoV‐2 in Nederland). Our dataset is not large enough to sufficiently calculate mortality rates, but the proportion of deceased patients (2/18 infected patients) implies a high risk of fatal outcome. Deceased patients had respiratory and/or cardiac comorbidities and were overweight. To put our results in perspective, we compared to the prevalence of COVID‐19 in the general Dutch population. The prevalence of COVID‐19 in patients with DM1 (9.2%) is lower than in the general Dutch population in the same period (11.5%), which was before October 5, 2021 (Epidemiologische situatie van SARS‐CoV‐2 in Nederland). This is probably the result of effective precautionary measures taken by DM1 patients. We found a similar hospitalization rate and prevalence compared to the general population, as was reported for another cohort of DM1 patients (Ilic Zivojinovic et al. 2023). The high rate of complicated COVID‐19 disease asks for effective precautionary measures in this vulnerable patient population.

Our survey data show a high rate of canceled standard care for DM1 patients. Reduction of standard care was mostly reported as a reduced number of appointments with specialists other than neurologists and physiotherapists, which potentially afflicts the multidisciplinary character of care for this patient population. Higher levels of anxiety, more pain/discomfort, and more limitations in daily activities negatively affect self‐perceived health status. Higher levels of fear/anxiety may be a direct effect of the COVID‐19 pandemic. DM1 patients are prone to be more negatively affected by the indirect effects of the pandemic, probably because of the common comorbidities (i.e., cognitive deficits, depression, apathy, and anxiety) among DM1 patients. Moderate stress levels in DM1 patients during the pandemic have been reported before (Eichinger et al. 2021). Regarding limitations of daily activities, during the COVID‐19 pandemic, a large proportion of day‐to‐day activities were limited by governmental restrictions. More pain/discomfort can be a result of indirect effects of the pandemic, for example, decreased physical activity due to “lockdown” restrictions.

Although we expected deterioration in EQ‐VAS scores in our DM1 patient population, as this was already reported in a healthy population during the COVID‐19 pandemic (Shah et al. 2021), this was not demonstrated in our DM1 population. We found that self‐reported activity limitations and participation restrictions, which are reflected in the DM1‐Activ^c^ score, were not different during the pandemic as compared to the time before. Also, the rate of change in DM‐Activ^c^ score remained unchanged during the pandemic. On the other hand, proxies reported a deterioration in mental well‐being and physical health of DM1 patients during the pandemic. The discrepancy between caregiver‐reported data and self‐reported data underlines the importance of the involvement of caregivers in the guidance of DM1 patients, as in clinical practice, it is often noted that DM1 patients show reduced awareness of the burden and progress of their disease. This was confirmed by a study showing that a large percentage (51.6%) of DM1 patients are unaware about the disease (Baldanzi et al. 2016).

All in all, in future similar situations, the implementation of social restrictions and downscaling of healthcare appointments in DM1 patients can be considered, whilst patients' proxies should be involved in the assessment of mental and physical well‐being of patients.

### 4.1 Limitations

Patients with asymptomatic COVID‐19 infection could be missed due to the absence of testing as this is not necessitated nor recommended, and antibody testing to track down past COVID‐19 infections was not feasible. Our sample size was limited to the patients included in the MYODRAFT database; a larger international cohort would provide more robust and generalizable results. Also, the number of patients with DM1‐Activ^c^ scoring during the pandemic was small, as many hospital appointments were canceled or were being converted to telephone consultations.

The additional COVID‐19 questionnaire was distributed shortly after the COVID‐19 pandemic. The retrospective nature of our study presents several limitations, including recall and temporal bias.

## 5 Conclusion

In conclusion, our study showed that the percentage of COVID‐19 infections in DM1 patients was lower than in the general population. DM1 patients were at risk for a more complicated course of disease when being infected with COVID‐19, with a high percentage (22.2%) of hospitalizations and symptomatic disease in all infected patients. In general, longitudinal patient‐reported data suggest no influence of the indirect effects of the COVID‐19 pandemic and its social restrictions on physical functioning and suggest similar strategies can be applied in future pandemics.

## Author Contributions

**Vera E. A. Kleinveld**: conceptualization, investigation, writing–original draft, methodology, formal analysis, project administration, data curation, visualization. **Johanna E. Bruijnes**: supervision. **Samuel Labrecque**: writing–review and editing, formal analysis. **Danielle E. G. Jeurissen‐Bekkering**: writing–review and editing. **Catharina G. Faber**: writing–review and editing. **Corinne G. C. Horlings**: writing–review and editing, supervision, methodology, conceptualization, resources.

## Conflicts of Interest

The authors declare no conflicts of interest.

### Peer Review

The peer review history for this article is available at https://publons.com/publon/10.1002/brb3.70162.

## Data Availability

The data that support the findings of this study are available from the corresponding author upon reasonable request.

## COVID‐19 Questionnaire

1 **General information**
  1.1 What is your age? (open field)
  1.2 What is your sex? (open field)
  1.3 What is your length? (open field)
  1.4 What is your weight? (open field)
  1.5 Did you receive a vaccine against the COVID‐19 virus? (yes/no)
    1.5.1 If yes—Which type of vaccine did you receive? (open field)
    1.5.2 If yes—How often did you receive a vaccination? (provide date of vaccinations)
2 **Consequences of the COVID‐19 pandemic**
  2.1 Starting from February 2020, did any appointments with a medical professional (e.g., a physician or a physiotherapist) get canceled because of COVID‐19? (yes/no)
    2.1.1 If yes—How many appointments were canceled and with whom? (general practitioner/neurologist/specialist other than neurologist/physiotherapist/others [open field])
    2.1.2 If yes—What were the reasons for cancellation? (canceled because I was infected with COVID‐19/canceled because I was afraid to get infected/canceled because I was in quarantine/others [open field])
  2.2 Since February 2020, you have suffered from symptoms for which you would normally make an appointment with a medical professional but have not done so? (yes/no)
    2.2.1 What type of medical professional? (open field)
    2.2.2 What were the reasons to not seek help from a medical professional? (open field)
  2.3 Since February 2020, did you receive new diagnoses?
    2.3.1 Please provide diagnosis and hospital name.
  2.4 Before the COVID‐19 pandemic, did you need (professional) care/support in daily life?
    2.4.1 If yes—How often?
    2.4.2 Did this change during the COVID‐19 pandemic? (yes/no)
      2.4.2.1 If yes—How did it change?
      2.4.2.2 If yes—How often do you receive professional care/support in daily life now?
3. **Mental well‐being**

Please indicate to what extent you agree with the following statements:

3.1 COVID‐19 has hindered my social life. (not/a little/a lot)
3.2 COVID‐19 has hindered me from performing my hobbies. (not/a little/a lot)
3.3 COVID‐19 has hindered me from performing my job. (not/a little/a lot)
3.4 I find the COVID‐19 virus threatening for my own health. (not/a little/a lot)
3.5 I find the COVID‐19 virus threatening for my social life. (not/a little/a lot)
3.6 I find the COVID‐19 virus threatening for my mental well‐being. (not/a little/a lot)
4. **Proxies (if available)**
  4.1 Indicate how you felt the patient was functioning physically before the COVID‐19 pandemic on a 0–10 scale. (0 = *worst*, 10 = *best*)
  4.2 Indicate how you felt the patient was functioning physically now on a 0–10 scale. (0 = *worst*, 10 = *best*)
  4.3 Indicate how good you thought the patient's mood was before the COVID‐19 pandemic on a 0–10 scale. (0 = *sad*, 10 = *most happy*)
  4.4 Indicate how good you thought the patient's mood was now on a 0–10 scale. (0 = *sad*, 10 = *most happy*)
5. **COVID‐19 infection**
  5.1 Did you ever receive a positive COVID‐19 test? (yes, confirmed by a doctor/no, but I did have symptoms/no)
    5.1.1 If yes, where did you receive this positive COVID‐19 test? (hospital/general practitioner/open field)
    5.1.2 If yes, when did you receive this positive COVID‐19 test? (date)
    5.1.3 If yes, how many days did you have symptoms related to COVID‐19? (it concerns symptoms you experienced within 14 days of the first symptom arising)
    5.1.4 From which COVID‐19‐related symptoms did you suffer? (fever/cough/sore throat/shortness of breath/cold/loss of taste or smell/muscle soreness/headache/fatigue/muscle weakness/gastro‐intestinal symptoms/increase in myotonic dystrophy symptom intensity/others [open field])
    5.1.5 Did you need a hospital admission because of a COVID‐19 infection?
      5.1.5.1 If yes—How many days were you hospitalized?
      5.1.5.2 If yes—On which department? (regular nursing ward/medium care/intensive care)
    5.1.6 Did you recover from your COVID‐19 infection? (yes/no, still recovering/no, still sick)
    5.1.7 What symptoms, arising after the COVID‐19 infection, do you currently still have? (fever/cough/sore throat/shortness of breath/cold/loss of taste or smell/muscle soreness/headache/fatigue/muscle weakness/gastro‐intestinal symptoms/increase in myotonic dystrophy symptom intensity/others [open field])
    5.1.8 Do you find that your symptoms of myotonic dystrophy have worsened after the COVID‐19 infection?
      5.1.8.1 If yes, what symptoms? (open field)
